# Mining Proteins with Non-Experimental Annotations Based on an Active Sample Selection Strategy for Predicting Protein Subcellular Localization

**DOI:** 10.1371/journal.pone.0067343

**Published:** 2013-06-26

**Authors:** Junzhe Cao, Wenqi Liu, Jianjun He, Hong Gu

**Affiliations:** 1 School of Control Science and Engineering, Dalian University of Technology, Dalian, China; 2 College of Information and Communication Engineering, Dalian Nationalities University, Dalian, China; Wageningen UR Livestock Research, The Netherlands

## Abstract

Subcellular localization of a protein is important to understand proteins’ functions and interactions. There are many techniques based on computational methods to predict protein subcellular locations, but it has been shown that many prediction tasks have a training data shortage problem. This paper introduces a new method to mine proteins with non-experimental annotations, which are labeled by non-experimental evidences of protein databases to overcome the training data shortage problem. A novel active sample selection strategy is designed, taking advantage of active learning technology, to actively find useful samples from the entire data pool of candidate proteins with non-experimental annotations. This approach can adequately estimate the “value” of each sample, automatically select the most valuable samples and add them into the original training set, to help to retrain the classifiers. Numerical experiments with for four popular multi-label classifiers on three benchmark datasets show that the proposed method can effectively select the valuable samples to supplement the original training set and significantly improve the performances of predicting classifiers.

## Introduction

A good understanding of protein subcellular location is a key for deducing protein functions, revealing disease pathogenesis, and identifying drag targets. In the last ten years, the rapid growth of protein data has made it faster and more economical to predict subcellular localization via computational methods. Since the first protein location prediction system emerged [1], many prediction approaches and predictors have been proposed. These methods are mostly based on classification algorithms, e.g. k-nearest neighbor (KNN) [2]–[5], support vector machine (SVM) [6]–[8], Bayesian methods [9], [10], and neural network [11], [12], etc. A comprehensive review [13] provides the process to establish a robust predictor of protein subcellular localization, with following aspects: (a) selecting or constructing an effective benchmark dataset to train and test the predictor; (b) formulating the protein samples with a valid mathematical expression; (c) proposing a powerful algorithm (classifier) for prediction tasks; and (d) performing proper tests to objectively evaluate the performance of the predictor. Among these aspects, one key factor of building a high-accuracy prediction method is to obtain a valid dataset with sufficient useful information to train a powerful classifier.

Normally, the training data of a subcellular localization predictor are acquired from the “proteins with experimental annotations (referred as PEAs hereafter)” in protein databases, which are labeled by sufficient experimental evidences. However, as we know, experimental methods require a long time to obtain conclusive evidence to assign an annotation. Therefore, these experimental protein sequences are just a small part of the overall sequences. According to the record (version 2012_05) of the central protein databank UniProtKB/Swiss-Prot, the PEAs only occupy 13.22% of the total reviewed protein contained therein. In this study, we also counted the number of the protein sequences over the past ten years in UniProtKB/Swiss-Prot and summarized the statistics in **Table 1**. Over the last decade, there was a tenfold increase in the amount of all protein sequences, but the growth of the experimental sequences was less than doubled. While more PEAs of all types are needed to provide useful information for increasing undetermined proteins, the gap between the amount of PEAs and the entire protein data are becoming larger and larger. In addition, for computational prediction methods, excess of homologous or similar protein data will cause the over-fitting problem and these data are redundant for training, consequently, most of these PEAs have to be abandoned in practice. Besides, some special subcellular locations are correlated with very few PEAs and it also restricts the number of data used for learning. Therefore, there are often insufficient PEAs when constructing a proper dataset for a prediction task. For instance, the virus benchmark dataset in paper [14] merely consists of 207 proteins, and there are only eight proteins located in “viral capsid”. The problem of lacking high-quality training data nearly occurs within each species and it has been a major problem in many bioinformatics researches because the prediction with sparse data would mostly obtain disappointing results [15].

**Table 1:** Number of protein sequences over the past ten years (2003–2012) in the UniProtKB/Swiss-Prot protein knowledgebase.

Release date	Database version	Total	PEAs	PNEAs
2003-12-15	1.0	135938	38903	45391
2004-07-05	2.0	148277	41031	50806
2005-05-10	5.0	178998	45606	65084
2006-10-31	9.0	239174	53510	94897
2007-07-24	12.0	274311	57490	113135
2008-07-22	14.0	390787	64733	167972
2009-09-01	15.7	495368	68029	220091
2010-07-13	2010_08	516934	70180	232546
2011-07-27	2011_08	531326	70552	241226
2012-05-16	2012_05	536029	70868	245342

The statistics is only from the UniProtKB/Swiss-Prot manually reviewed entries, and the unreviewed entries in the UniProtKB/TrEMBL are not included.

To overcome this shortage of training data, seeking extra protein training data becomes a very natural idea. Besides the PEAs, we recently find that we can take advantage of the huge number of “proteins with non-experimental annotations (referred as PNEAs hereafter)” in the central protein database UniProtKB/Swiss-Prot. Since the observations are not marked from direct experiments, non-experimental annotations are labeled based on non-experimentally proven findings such as logical or conclusive evidences, sequence analysis results, biological events and characteristics [16]. A PNEA has at least one non-experimental label in its “Subcellular location” item, and a non-experimental label corresponds to one of the following three types [17]: “Probable” - from non-direct experimental evidences; “Potential” - from computer prediction, logical or conclusive evidences; “By similarity” - from experimental evidences in a close member of the family. The details of the three non-experimental labels can be found in the UniProtKB/Swiss-Prot manual at http://www.uniprot.org/manual. For protein subcellular location prediction based on computational methods, the PNEAs who are being ignored are important and valuable. Unlike unknown protein data, the PNEAs provide a lot of high reliable reference location information. Additionally, as shown in **Table 1**, PNEAs have a much larger number and grow much faster than PEAs. If such abundant PNEAs can be effectively exploited, they would provide a huge supplement to PEAs for training more powerful predictors. Despite the big advantage of PNEAs, not all of them can be indiscriminately used as supplementary training data. The reason is that the non-experimental evidence is still weaker than the experimental proof, so some portion of PNEAs may have inaccurate non-experimental labels. Therefore, a feasible rule is needed to select the useful members of the PNEAs with a low risk and high quality for training a classifier.

In order to develop a proper rule for the active selection process, a machine learning technique named “active learning” is adopted in our study. This active learning method is a paradigm for using unlabeled data to complement labeled data, as it can actively select and learn from the most informative unlabeled data. The idea of actively selecting new samples is suitable for our work. However, there are some issues with the active learning process that need to be resolved before it can be properly used in this study. The active learner always actively asks the user to label the unlabeled data so that it can learn a good classifier with as few manual labeled samples as possible [18]; while in our study, the candidate PNEA samples are not unlabeled but rather have special non-experimental labels, and the proposed algorithm should automatically pick out enough but not redundant samples from the whole PNEA dataset. Therefore, inspired by an active learning algorithm [19], this paper proposes such a novel active sample selection strategy for PNEAs to increase the amount of training data available. For the weak basic classifiers learned via only the original data, this strategy measures the usefulness of all candidate PNEAs, and picks out these most useful PNEAs as supplementary training data. The weak classifiers are then retrained on the new training set to obtain improved prediction performances.

The effectiveness of the proposed approach is tested on three protein benchmark datasets from virus, plant and gram-negative bacteria cells, by four popular multi-label learning classification algorithms which are based on KNN, SVM, Bayesian method and neural network. The results show that the proposed method can effectively pick out the useful PNEAs and there are obvious enhancements for the prediction performances of each basic classifier.

## Materials and Methods

### The Datasets

Three existing benchmark experimental datasets of different species are used for cross-validation tests, which include a virus dataset [14] consisting of 207 proteins and 6 different subcellular location classifications, a plant dataset [20] consisting of 978 proteins and 12 different subcellular location classifications, and a Gram-negative bacteria (referred as Gneg hereafter) dataset [21] consisting of 1392 proteins and 8 different subcellular location classifications. In order to obtain effective candidates for supplementary training data, we extracted numerous PNEAs of the three species by parsing the “Subcellular location” section of the “Comments” field in UniProtKB/Swiss-Prot database (release 2012_05). Protein fragments and those containing less than 50 amino acid residues were discarded. Similarly, we also collected several new PEAs which were not included in the above-mentioned benchmark datasets for an independent test. In order to reduce the redundancy and avoid homology bias, we used a public server PISCES [22] based on PSI-BLAST alignments to identify and cull protein sequences from all the sequence data extracted to ensure that none of these proteins have a ≥25% sequence similarity to one another as well as any sequence in the benchmark dataset for the same species.

After culling, we created three supplementary training sample pools as candidates for active selection, which consist of 238 virus PNEAs, 758 plant PNEAs and 248 Gneg PNEAs. We also constructed three additional independent test sets, consisting of 69 virus PEAs, 261 plant PEAs and 207 Gneg PEAs. Note that, because some proteins occur in more than one location, the concept of “locative protein” in the literature [21] is employed to compute performance indexes of the classifiers. This concept means that a protein coexisting at *N* (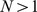) different location sites will be counted as *N* locative proteins even if they have an identical sequence. The amounts of active/locative proteins in the three groups of datasets are shown in **Table 2**. More details about the datasets can be found in **Table S1–S3** in Material S1. The new PNEAs and new PEAs used in our research are all listed in Material S2 (**Supplementary Dataset S1–S6**).

**Table 2:** Number of active/locative proteins in the three groups of datasets.

Dataset	Number of classes	benchmark datasets [14], [20], [21]	Supplementary training sample pool	Independent test set
Virus	6	207/252	238/289	69/93
Plant	12	978/1055	758/813	261/301
Gneg	8	1392/1456	248/271	207/225

### Active Sample Selection Strategy

In this study, because some proteins have multiple subcellular localization sites, the final prediction task is also a multi-label learning problem. Accordingly, the active sample selection strategy should have the ability to deal with the multi-label cases. Let 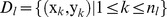 denote the original training data set consisting of 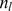 PEAs classified in 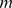 different subcellular locations, where each protein 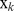 can be represented by a feature vector of 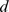 dimensions as 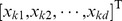, and the label set 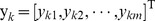 denotes the protein subcellular locations of 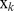. For each protein 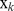, if it inhabits the 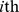 subcellular location, mark 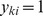, otherwise 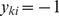. The basic classifier 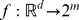 is trained by 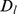 to output a set of labels for each unseen protein. Let 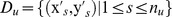 denote the supplementary training sample pool containing 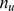 PNEAs, where a protein 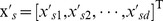 has the label set 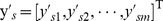. For each protein 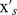, if it has the 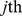 subcellular location labeled by experimental/non-experimental evidences, we mark that 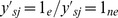, otherwise 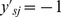. Note that, both 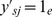 and 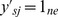 mean 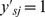, and the subscripts are merely used for recognizing that this positive label is obtained by corresponding experimental or non-experimental annotations.

In order to actively pick out the useful samples from the supplementary training sample pool, the key is to create a feasible evaluation function to measure the usefulness of a non-experimental sample and decide which samples should be added into the original training set. In this paper, the classification risk of a sample is used for reflecting the sample’s usefulness, where a lower risk means a higher usefulness. For a sample 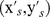 in 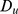, let 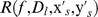 be the classification risk which is brought by adding 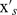 into the original training set, and the evaluation function 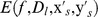 of 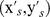 is defined by its maximum risk. Our motivation is to evaluate the risks, and pick out the optimal 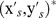 by minimizing the maximum risk, that leads to the following min-max combinatorial optimization problem:
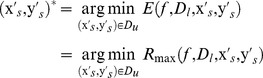
(1)

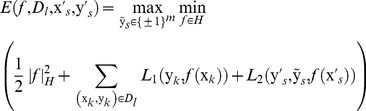
(2)where, 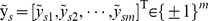 represents the unknown actual label set for 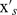, where, for each label 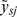, 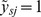 if 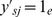, 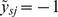 if 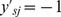, but if 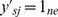 then 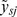 may be 1 or −1. 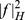 is the regularization item which measures the model complexity of the classifier, here 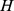 is a reproducing kernel Hilbert space endowed with kernel function 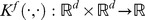. 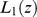 is a quadratic loss function and 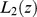 is a weighted quadratic loss function, i.e.,
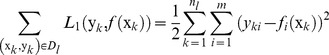
(3)

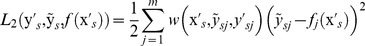
(4)


where, 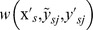 is the weight function. For a PNEA, its associated label set is uncertain because its non-experimental label may not be the active label, and it is hard to directly calculate its loss. Therefore, the weight function 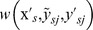 is added to reflect the probability that a non-experimental label is the active label, which can be written as:
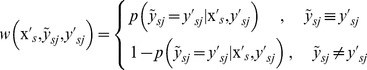
(5)here 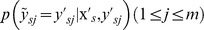 is the posterior probability of the event that 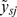 just equals 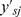 when 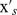 has a non-experimental label 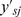. According to the previous description of 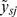, it can be deduced that 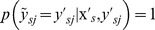 when 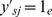 or 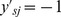. Therefore, it only needs to estimate the posterior probability for a non-experimental label 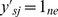. We use the Parzen-window estimation with the Gaussian kernel [23] to estimate the posterior probability of 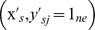 as:
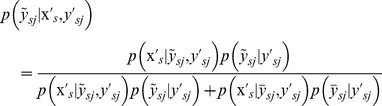
(6)where, the prior probability 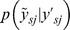 is the confidence of the event that if 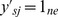 then 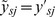, and it is set as the parameter related to the type of the corresponding non-experimental label 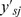, 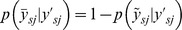 is the complementary set of 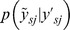, 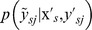 and 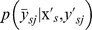 are short for 
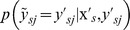
 and 
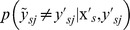
 respectively, which are defined as:
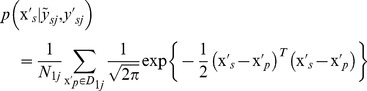
(7)

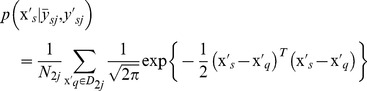
(8)


where, 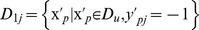 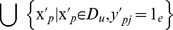 consisting of 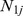 samples is the set of all samples with certain labels; 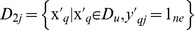 consisting of 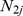 samples defined as the set of all samples with non-experimental labels because a PNEA sample will get the maximum loss when all the actual label are opposite to the corresponding non-experimental positive label, i.e. 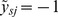 and 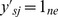.

Plugging equations (3)-(5) into equation (2), this active sample selection can be written as following the min-max optimization problem:
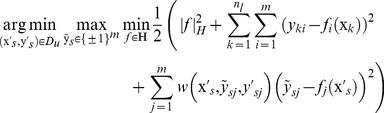
(9)


From the derivation, we have:
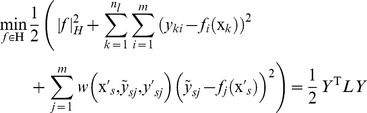
(10)where, 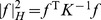, 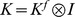, 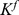 is the kernel matrix of size 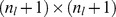, 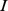 is an 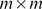 identity matrix, 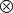 is the Kronecker product, 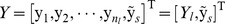, 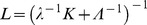, and
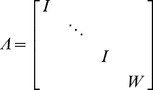
(11)

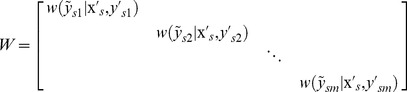
(12)


Thus, the evaluation function 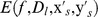 is simplified as:
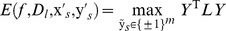
(13)


Let 
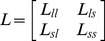
, then
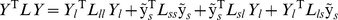
(14)


Except for 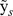, all other parts in Eq.(14) can be determined and the min-max optimization problem described as Eq.(8) can be solved through using all feasible values of 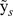 to find the optimal 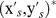 with the smallest 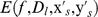. Similarly, we can pick out other PNEA samples one by one.

Since the usefulness of all the PNEAs are being measured, the algorithm needs to decide how many samples in 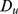 should be added to 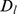 to help to retrain the classifier. We observe that there is a high correlation between the usefulness of PNEA samples in 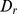 and the change rates of the evaluation values. If the change becomes stable, it means the latest added supplementary training samples have little or no effect. Based on this point, this paper presents a simple algorithm, which can output a proper proportion of all samples in the supplementary training sample pool. First, rank all the samples within 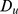 in ascending order according to their evaluation to compose a new ordered set 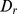. Next, denote the evaluation value of a sample 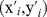 in 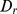 by 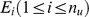. Then the change rate of its evaluation value 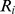 can be written as:
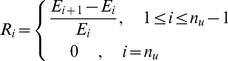
(15)


For a given step of proportion 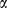 and the corresponding number of intervals 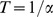, the algorithm needs to decide which proportion is preferred for helping to retrain the basic classifier (e.g. 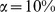, then 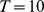, where the preferred proportion is one of following percentages: 10%, 20%, 30%, …, and 100%). Let 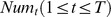 be the number of the samples in the *t*-th interval, and the preferred proportion 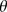 can be calculated as:
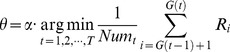
(16)

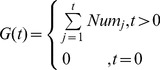
(17)


Note that, it is hard to theoretically prove whether the output proportion 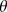 is the global optimum or not, but it can be seen that 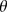 can indeed provide excellent results in subsequent simulation experiments.

**Figure 1:**
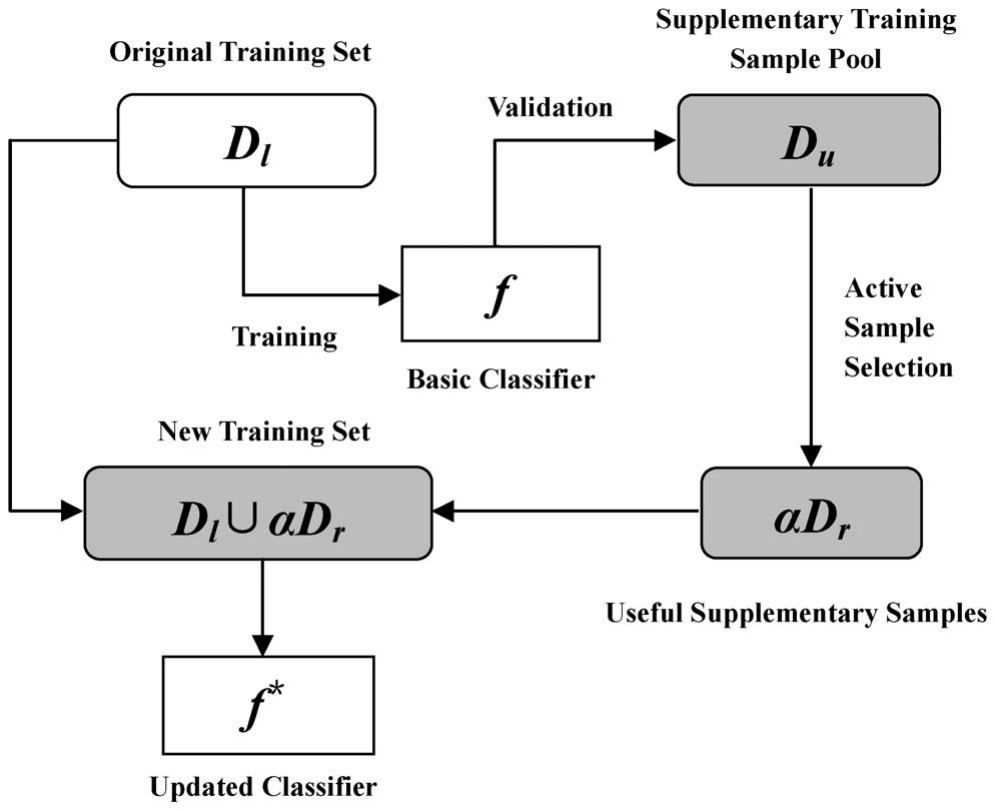
The work process of the proposed active sample selection strategy.

After selecting the top 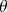 of the samples in 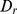 and adding them into the original training set, the initial classifier is updated according to the new training set and its performance is improved. An illustration of the work process of the proposed active example selection strategy is shown in Fig. 1.

### Evaluation Metrics

In order to comprehensively evaluate the active sample selection method and compare the classifier performances with/without the proposed approach, some common evaluation metrics are used. Here, 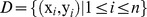 denotes a test set, 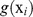 returns a set of proper labels of 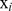; 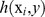 returns a probability indicating the confidence for 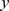 to be a proper label of 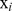; 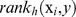 is the rank of 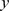 derived from 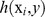. Let 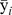 and 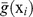 represent the complementary sets of 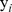 and 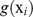 , respectively. Therefore, we have:

- True Positives: 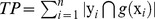

- False Positives: 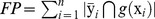

- True Negatives: 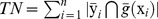

- False Negatives: 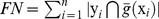

Based on the above, three global indices: accuracy (Accu), Matthews correlation coefficient (MCC) and F1-scroe, and three multi-label evaluation metrics: average precision (Avgprec), ranking loss (Rloss) and coverage are computed as follows:
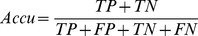
(18)

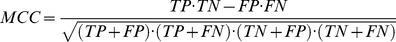
(19)

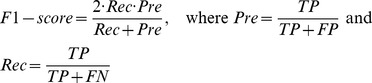
(20)

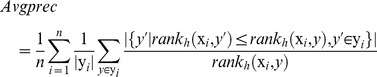
(21)

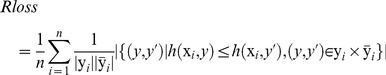
(22)

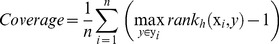
(23)


## Results and Discussion

We performed several simulation experiments to evaluate the performance of the proposed approach through both the sub-sampling (10-fold cross validation) and independent dataset test methods using the three groups of datasets mentioned in section “Material and Methods”. In the sub-sampling tests, we performed multiple rounds of randomizations of the original training and testing data on each benchmark dataset. In the independent dataset tests, the benchmark datasets were directly used as the original training sets, and the new independent test sets were adopted for testing. The amphiphilic pseudo amino acid composition [24] was employed as the feature extraction technology to represent a protein sequence. The protein sequences were formulated with a valid mathematical expression by this method through a public online server named PseAAC at: http://www.csbio.sjtu.edu.cn/bioinf/PseAA/. The details of PseAAC can be found in reference [25]. In this study, amino acid characters were empirically chosen to be Hydrophobicity, Hydrophilicity and Mass; the weight factor was 0.4, and the lambda parameter was 5. Four different types of multi-label classification models including IMKNN [5], SVM [26], Gaussian process [10] and ML-RBF [27], were used as basic classifiers to test our algorithm. The parameters of these classifiers were assigned the same values as the original papers and all these parameters were fixed in the whole experiments for an objective comparison.

The overall performances of the above classification algorithms following three kinds of conditions were compared. These conditions were: not using the proposed active sample selection (using no supplementary training samples), using the proposed active sample selection with a preferred proportion (top 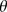) of the supplementary training samples, and directly using the whole PNEA samples in the supplementary training sample pool. In the experiments, the kernel function of 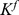 was the same Gaussian kernel used for estimating the posterior probability in Eq.(6). The prior probabilities of the three levels of non-experimental labels were set according to the strength of the evidences of the three non-experimental label types: the prior probability with “Probable” label was set to be the largest, the value of “Potential’ was medium and “By Similarity” was the smallest. We tested several values for the prior probabilities and finally choose a group of values with the best results as: 0.85 for “Probable”, 0.8 for “Potential” and 0.75 for “By Similarity”. The step of proportion 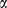 was set to 10% and the number of intervals 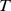 was 10.

Through the numerical experiments, we observe the preferred proportions of active sample selection for various datasets are different. The preferred proportion of virus PNEA samples is 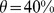, 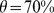 for plant, and 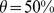 for Gram-negative bacteria. The comparisons of the performances of these classification models by using none, preferred proportion and all of the samples in the supplementary training sample pool are shown in **Table 3** **,** **Table 4** **,** **Table 5** **,** **Table 6**. **Table 3** **, **Table 4** and** **Table 5** show the average values of 10 randomizations, 10-fold cross-validation measures and their standard deviations, and **Table 6** shows the results of the independent dataset test. For each evaluation metric, “↑” means the bigger the metric value the better the performance, and “↓” means the smaller the metric value the better the performance. It can be seen, for each case, the classifier using the supplementary training data selected by the proposed approach always performs better than the basic classifier using no supplementary training sample. Additionally, the results under the proposed approach are superior to that of indiscriminately using the whole data in the supplementary training data pool. From the simulation results, it can be concluded that, on one hand, the improvements to the original prediction indicates that the selected PNEA samples are useful and indeed provide helpful information for prediction; on the other hand, the better performance of the active sample selection over directly using all the samples in the supplementary training sample pool indicates that a part of the PNEA samples disrupts the prediction because they may have some inaccurate information. Therefore, an effective active sample selection is important to select a proper amount of valuable PNEA samples and reduce the possibility of prediction disturbance brought by the redundant supplementary training data. We also observed that the performance improvements of all the classification models are related to the size of the original training set. For the virus cases with the least original training data, each classifier’s performance improvement is superior to that of the other two datasets. On the contrary, for the Gneg cases with the most original training data, the improvement effect is the smallest. We attribute this fact to the original training set with less data having a greater data shortage, so the basic classifiers are better improved by incrementally adding useful supplementary training data. Without dependence on the original classification model, the experiment results show that the proposed active sample selection strategy provides a generic approach for the existing prediction algorithms.

**Table 3:** Results for different basic classifiers (mean±SD) by using varied numbers of supplementary training data, trained and tested in 10-fold cross-validation on the virus dataset.

Classifier	Ealuation metrics	Number of supplementary training data		
		None	Top 40%	All
IMKNN	Accu↑	0.7753±0.0257	**0.8041±0.0252**	0.7944±0.0369
	MCC↑	0.2796±0.0515	**0.3889±0.0478**	0.3600±0.0484
	F1-score↑	0.4131±0.0674	**0.5179±0.0539**	0.4886±0.0584
	Avgprec↑	0.5978±0.0596	**0.6559±0.0507**	0.6502±0.0541
	Rloss↓	0.6126±0.0147	**0.5036±0.0149**	0.5276±0.0161
	Coverage↓	1.6591±0.3007	**1.5269±0.2550**	1.5555±0.2919
SVM	Accu↑	0.7855±0.0199	**0.8059±0.0218**	0.7887±0.0381
	MCC↑	0.3432±0.0581	**0.3952±0.0471**	0.3739±0.0426
	F1-score↑	0.4758±0.0457	**0.5160±0.0526**	0.5070±0.0676
	Avgprec↑	0.6385±0.0436	**0.6752±0.0481**	0.6553±0.0419
	Rloss↓	0.5376±0.0222	**0.4915±0.0268**	0.5112±0.0224
	Coverage↓	1.5376±0.1366	**1.4795±0.2242**	1.5384±0.2237
Gaussian process	Accu↑	0.7979±0.0224	**0.8220±0.0127**	0.7991±0.0286
	MCC↑	0.3382±0.0520	**0.4026±0.0359**	0.3430±0.0437
	F1-score↑	0.4543±0.0548	**0.5816±00390**	0.4616±0.0481
	Avgprec↑	0.6147±0.0228	**0.6477±0.0230**	0.6332±0.0233
	Rloss↓	0.5989±0.0298	**0.5508±0.0295**	0.5688±0.0201
	Coverage↓	1.5917±0.1892	**1.5404±0.1588**	1.5651±0.1946
ML-RBF	Accu↑	0.6783±0.0224	**0.7517±0.0213**	0.7421±0.0208
	MCC↑	0.2749±0.0269	**0.3505±0.0161**	0.3378±0.0239
	F1-score↑	0.3720±0.0400	**0.4436±0.0203**	0.4103±0.0369
	Avgprec↑	0.5751±0.0595	**0.6215±0.0453**	0.5938±0.0469
	Rloss↓	0.3968±0.0135	**0.3194±0.0208**	0.3760±0.0189
	Coverage↓	2.2487±0.3568	**1.8906±0.2886**	2.1721±0.3477

**Table 4:** Results for different basic classifiers (mean±SD) by using varied numbers of supplementary training data, trained and tested in 10-fold cross-validation on the plant dataset.

Classifier	Ealuation metrics	Number of supplementary training data		
		None	Top 50%	All
IMKNN	Accu↑	0.8557±0.0058	**0.8917±0.0035**	0.8881±0.0045
	MCC↑	0.1362±0.0254	**0.1636±0.0277**	0.1498±0.0285
	F1-score↑	0.1858±0.0210	**0.2124±0.0245**	0.1903±0.0253
	Avgprec↑	0.2943±0.0153	**0.3103±0.0168**	0.2934±0.0175
	Rloss↓	0.8523±0.0178	**0.8333±0.0152**	0.8546±0.0123
	Coverage↓	5.9920±0.2188	**5.7544±0.2598**	5.9703±0.2037
SVM	Accu↑	0.8742±0.0050	**0.8820±0.0057**	0.8804±0.0081
	MCC↑	0.2215±0.0288	**0.2649±0.0221**	0.2529±0.0232
	F1-score↑	0.2904±0.0261	**0.3294±0.0292**	0.3183±0.0288
	Avgprec↑	0.4049±0.0151	**0.4331±0.0246**	0.4271±0.0439
	Rloss↓	0.7114±0.0262	**0.6777±0.0336**	0.6871±0.0383
	Coverage↓	4.9945±0.2491	**4.7985±0.2170**	4.8574±0.2372
Gaussian process	Accu↑	0.8909±0.0013	**0.9116±0.0045**	0.9096±0.0031
	MCC↑	0.2084±0.0287	**0.2421±0.0178**	0.2132±0.0135
	F1-score↑	0.1796±0.0284	**0.2218±0.0153**	0.1963±0.0211
	Avgprec↑	0.2884±0.0227	**0.3125±0.0184**	0.2934±0.0135
	Rloss↓	0.8878±0.0216	**0.8559±0.0279**	0.8769±0.0141
	Coverage↓	5.8800±0.2047	**5.7248±0.2623**	5.8947±0.2497
ML-RBF	Accu↑	0.8803±0.0084	**0.8994±0.0046**	0.8898±0.0031
	MCC↑	0.2663±0.0177	**0.2705±0.0200**	0.2656±0.0234
	F1-score↑	0.3211±0.0161	**0.3332±0.0162**	0.3230±0.0230
	Avgprec↑	0.5511±0.0261	**0.5682±0.0159**	0.5526±0.0192
	Rloss↓	0.2356±0.0160	**0.2211±0.0216**	0.2301±0.0164
	Coverage↓	2.7591±0.1611	**2.5926±0.2111**	2.6839±0.1984

**Table 5:** Results for different basic classifiers (mean±SD) by using varied numbers of supplementary training data, trained and tested in 10-fold cross-validation on the Gram-negative bacteria dataset.

Classifier	Ealuation metrics	Number of supplementary training data		
		None	Top 70%	All
IMKNN	Accu↑	0.8699±0.0073	**0.8819±0.0063**	0.8688±0.0073
	MCC↑	0.5437±0.0233	**0.5527±0.0290**	0.5452±0.0225
	F1-score↑	0.6092±0.0193	**0.6170±0.0184**	0.6079±0.0227
	Avgprec↑	0.6894±0.0152	**0.7269±0.0183**	0.7152±0.0198
	Rloss↓	0.2910±0.0243	**0.2792±0.0299**	0.3055±0.0254
	Coverage↓	1.4717±0.1886	**1.4345±0.1331**	1.4828±0.1173
SVM	Accu↑	0.9026±0.0067	**0.9062±0.0013**	0.9056±0.0034
	MCC↑	0.5698±0.0281	**0.5847±0.0147**	0.5828±0.0119
	F1-score↑	0.6258±0.0242	**0.6390±0.0128**	0.6366±0.0156
	Avgprec↑	0.7193±0.0157	**0.7210±0.0143**	0.7199±0.0117
	Rloss↓	0.3700±0.0191	**0.3575±0.0132**	0.3631±0.0164
	Coverage↓	1.7672±0.0698	**1.7003±0.0665**	1.7115±0.0672
Gaussian process	Accu↑	0.9332±0.0035	**0.9384±0.0042**	0.9300±0.0057
	MCC↑	0.6678±0.0106	**0.6878±0.0142**	0.6666±0.0171
	F1-score↑	0.6990±0.0263	**0.7096±0.0203**	0.6984±0.0156
	Avgprec↑	0.7307±0.0256	**0.7417±0.0220**	0.7264±0.0189
	Rloss↓	0.3722±0.0248	**0.3682±0.0238**	0.3728±0.0207
	Coverage↓	1.7689±0.0581	**1.7191±0.0635**	1.7989±0.0732
ML-RBF	Accu↑	0.9159±0.0035	**0.9319±0.0071**	0.9127±0.0015
	MCC↑	0.6144±0.0187	**0.6328±0.0183**	0.6020±0.0117
	F1-score↑	0.6672±0.0168	**0.6945±0.0128**	0.6507±0.0113
	Avgprec↑	0.8057±0.0145	**0.8295±0.0153**	0.7984±0.0086
	Rloss↓	0.1147±0.0120	**0.1044±0.0178**	0.1110±0.0067
	Coverage↓	0.8786±0.0503	**0.8499±0.0580**	0.8687±0.0538

**Table 6:** Comparison of the prediction results of different basic classifiers by using varied numbers of supplementary training data.

Dataset	Ealuation metrics	Number of supplementary training data												
		IMKNN			SVM			Gaussian process			ML-RBF			
		None	Top *θ*	All	None	Top *θ*	All	None	Top *θ*	All	None	Top *θ*	All	
Virus	Accu↑	0.7476	**0.7696**	0.7427	0.7476	**0.7672**	0.7451	0.7525	**0.7784**	0.7672	0.6397	**0.7574**	0.7328	
	MCC↑	0.2518	**0.3353**	0.2604	0.2518	**0.3257**	0.2589	0.1318	**0.2634**	0.2095	0.1255	**0.2919**	0.1957	
	F1-score↑	0.4114	**0.4835**	0.4262	0.4114	**0.4751**	0.4222	0.2406	**0.3662**	0.3166	0.3581	**0.4469**	0.3626	
	Avgprec↑	0.5572	**0.5982**	0.5438	0.5572	**0.5902**	0.5431	0.4480	**0.5217**	0.5008	0.4994	**0.6061**	0.5757	
	Rloss↓	0.6380	**0.5654**	0.6390	0.6380	**0.5713**	0.6419	0.8415	**0.7245**	0.7485	0.4931	**0.3326**	0.3622	
	Coverage↓	2.2059	**2.0441**	2.2941	2.2059	**2.0735**	2.3088	2.5735	**2.5294**	2.5441	3.0000	**2.0147**	2.2941	
Plant	Accu↑	0.9061	**0.9103**	0.9074	0.9042	**0.9087**	0.9068	0.9081	**0.9112**	0.9090	0.7350	**0.7982**	0.7816	
	MCC↑	0.3906	**0.4107**	0.3991	0.4220	**0.4410**	0.4325	0.2344	**0.2924**	0.2636	0.1428	**0.1908**	0.1531	
	F1-score↑	0.4346	**0.4501**	0.4423	0.4737	**0.4893**	0.4823	0.1864	**0.2527**	0.2276	0.2441	**0.2818**	0.2516	
	Avgprec↑	0.5099	**0.5142**	0.5121	0.5762	**0.5819**	0.5784	0.2971	**0.3359**	0.3222	0.4367	**0.4622**	0.4266	
	Rloss↓	0.6040	**0.5975**	0.6001	0.5212	**0.5136**	0.5200	0.8819	**0.8309**	0.8478	0.3670	**0.3372**	0.3722	
	Coverage↓	4.4636	**4.4291**	4.4674	3.8867	**3.8314**	3.8812	6.2222	**6.0153**	6.0498	4.4598	**4.0728**	4.5402	
Gneg	Accu↑	0.8374	**0.8441**	0.8386	0.8823	**0.8829**	0.8811	0.8950	**0.8981**	0.8932	0.8811	**0.8932**	0.8732	
	MCC↑	0.4542	**0.4683**	0.4607	0.4913	**0.4970**	0.4918	0.4924	**0.5100**	0.4777	0.4399	**0.4839**	0.4008	
	F1-score↑	0.5315	**0.5429**	0.5366	0.5591	**0.5650**	0.5605	0.5362	**0.5532**	0.5191	0.5000	**0.5294**	0.4655	
	Avgprec↑	0.6291	**0.6340**	0.6218	0.6331	**0.6402**	0.6361	0.5722	**0.5847**	0.5572	0.6935	**0.7267**	0.7011	
	Rloss↓	0.3891	**0.3743**	0.3812	0.4481	**0.4342**	0.4391	0.5503	**0.5343**	0.5707	0.1715	**0.1555**	0.1700	
	Coverage↓	2.3010	**2.1893**	2.2476	2.6165	**2.5388**	2.5680	3.0291	**2.9417**	3.1214	1.3204	**1.2136**	1.3155	

It is worth noting that, the inherent problem with PNEAs is that they can only be experimentally validated. To validate that the proposed strategy is more useful than conventional analysis based simply on PEA, it is better to test it via additional biological experiments. If we can show on PNEA data that the strategy finds true positives and rejects true negatives validated against biological observation of the characteristics of these proteins, the effectiveness of this approach will be further verified. However, in our work, it is difficult to directly conduct biological experiments to validate PNEAs. In a different way, we tried to find true positive and true negative PNEA samples which can be validated against a biological observation in the Swiss-Prot databank. Unfortunately, we found few true positives (e.g. the non-experimental annotation “Golgi apparatus” of the plant protein with entry number “Q9M2T1” has been verified experimentally) and no true negatives. Although the true positives can be successfully found using this strategy, we think the amount of samples identified is too small to provide enough support for this study. Therefore, the related results of these few protein samples are not included in this paper. Moreover, the objective of this study is not to identify true positive proteins, but to make protein subcellular localization prediction tools with better performance in accuracy with the help of non-experimental proteins. According to the results in Table **Table 3** **,** **Table 4** **,** **Table 5** **,** **Table 6**, the increase in accuracy over the conventional algorithms after training with these PNEAs indicates the proposed strategy works. Therefore, the proposed method could be thought of as potentially significant, even without the experimental biological validation. However, it is still worth to perform a biological validation for our algorithm, and we hope to cooperate with biochemists to improve this method in the future.

In summary, in order to overcome the shortage of experimental training data in the prediction of protein subcellular location, we mined the proteins with non-experimental annotations and designed a novel active sample selection strategy to find useful PNEA samples. As supplementary training data, these selected samples helped retrain and improve the original basic classifiers. This approach based on the min-max view provides a systematic way for measuring the usefulness of a sample with multiple labels. From the results, it can be clearly seen that the proposed algorithm is significant and valid to increase the predicting performance of all four types of classifiers. We believe that active sample selection techniques in machine learning can be used as a powerful and useful tool to alleviate the data shortage problem and it could be extended to other real-world data mining applications. We also expect that the information of a huge number of proteins with non-experimental annotations can be applied to other biological problems. Furthermore, in order to make the presented method available to compare with the predictors by other interested users, we will make efforts to provide an online prediction web-server with practical value in our future work.

## Supporting Information

Material S1 **This includes Tables S1–S3.**
(PDF)

Material S2 **This includes Datasets S1-S6.**
(PDF)
