# Supplementary material for: Mining Proteins with Non-Experimental Annotations Based on an Active Sample Selection Strategy for Predicting Protein Subcellular Localization
Source: PLoS One. 2013 Jun 26;8(6):e67343. doi: 10.1371/journal.pone.0067343 (PMC3694045; doi:10.1371/journal.pone.0067343)
Supplement: Material S1 — This includes Tables S1–S3. (PDF) [file pone.0067343.s001.pdf]

Table S1. Number of virus locative proteins

Subcellular location	Original training set	Supplementary training sample pool	Independent test set
Viral capsid	8	2	1
Host cell membrane	33	11	25
Host endoplasmic reticulum	20	18	17
Host cytoplasm	87	116	24
Host nucleus	84	113	24
Secreted	20	29	2
Over all	252	289	93

For the original training set consisting of 207 different proteins, 165 belong to one subcellular location, 39 to two locations, and 3 to three locations; for the supplementary training data pool consisting of 238 different proteins, 195 belong to one subcellular location, 37 to two locations, 5 to three locations, and 1 to five locations; for the independent test set consisting of 69 different proteins, 47 belong to one subcellular location, 20 to two locations, and 2 to three locations.

Table S2. Number of plant locative proteins

Subcellular location	Original training set	Supplementary training sample pool	Independent test set
Cell membrane	56	6	46
Cell wall	32	22	2
Chloroplast	286	159	49
Cytoplasm	182	146	37
Endoplasmic reticulum	42	6	28
Extracellular	22	24	6
Golgi apparatus	21	16	19
Mitochondrion	150	174	27
Nucleus	152	245	61
Peroxisome	21	5	4
Plastid	39	1	7
Vacuole	52	9	15
Overall	1055	813	301

For the original training set consisting of 978 different proteins, 904 belong to one subcellular location, 71 to two locations, and 3 to three locations; for the supplementary training data pool consisting of 758 different proteins, 703 belong to one subcellular location, and 55 to two locations; for the independent test set of 261 different proteins, 223 belong to one subcellular location, 36 to two locations, and 2 to three locations.

Table S3. Number of Gram-negative bacterium locative proteins

Subcellular location	Original training set	Supplementary training sample pool	Independent test set
Cell inner membrane	557	22	72
Cell outer membrane	124	25	39
Cytoplasm	410	9	47
Extracellular	133	41	47
Fimbrium	32	19	6
Flagellum	12	3	2
Nucleoid	8	7	1
Periplasm	180	145	11
Overall	1456	271	225

For the original training set consisting of 1392 different proteins, 1328 belong to one subcellular location, and 64 to two locations; for the supplementary training data poll consisting of 248 different proteins, 229 belong to one subcellular location, 15 to two locations, and 4 to three locations; for the independent test set consisting of 207 different proteins, 189 belong to one subcellular location, and 18 to two locations.
