# Supplementary material for: Mining Proteins with Non-Experimental Annotations Based on an Active Sample Selection Strategy for Predicting Protein Subcellular Localization
Source: PLoS One. 2013 Jun 26;8(6):e67343. doi: 10.1371/journal.pone.0067343 (PMC3694045; doi:10.1371/journal.pone.0067343)
Supplement: Material S2 — This includes Datasets S1-S6. (PDF) [file pone.0067343.s002.pdf]

Dataset S1. The virus PNEA entries for training including 238 different proteins

Q8JJX1 P13200 Q66642 P89905 P03248 P14998 P28982 Q0Q474 Q68749 Q8QL52 Q08595 A0MD33 Q00848 P28993 P20954 Q02122 Q07520 A0MD32 P28992 P05769 B1PS79 P27333 P28994 P16483 P16652 P22171 Q07631 P15098 P36324 Q77JX3 Q913Y8 Q2HR75 O42083 Q08454 O72736 P69616 Q91J26 Q86523 B1PS77 P09269 D5LX59 P16089 Q0H8C2 P31628 P68446 A2T3M2 O91251 Q5MKM7 Q5GA89 Q9E787 Q82855 P0C8K2 Q9JGU1 P49303 Q8JP02 P23430 P14547 O72121 Q6UDI6 Q05054 Q9G053 P33829 P29249 P13418 P0CA44 P54636 Q65474 P11335 Q91QN9 P26625 O36635 Q2Y2M6 P12923 P31332 P23628 P0C794 P0C8F4 Q6XDK7 A4PBQ0 A8Y986 Q8AZM0 P48309 P13895 P0C9D6 O55519 O42038 P28960 P15911 P25073 P24378 Q2HR64 P12366 A4PBP6 Q9E784 P19192 Q6DN64 Q0PI70 O41965 Q77SJ8 Q86112 Q8B912 Q18LD3 Q9JGU0 Q08453 P03525 P12472 P89467 Q6UDL8 P04935 P12325 O36972 Q91DS2 P21046 P28889 P14553 Q9E6P7 Q9IVZ8 P04877 P28888 A4PBP9 Q6UDK6 P0C979 P17380 Q288N6 Q6UDG0 P16725 O70790 P35341 P09263 Q01769 P68576 P17381 O11453 Q86134 Q9E6M8 Q9E7N7 P52340 Q5UQG2 Q66636 Q01008 P01104 Q66646 P52530 P03073 P10187 Q6UDJ5 O89749 Q3KSU8 P30020 P16726 O10440 P09245 P09300 P09276 Q87013 P07297 Q91FF5 P28932 Q6UDF2 P09303 P03187 P28955 P52348 P03254 Q6UDK7 P40628 Q80GM6 P29169 P27558 P17147 P07918 P89474 Q89707 P54087 P24441 P01102 Q6UDI9 Q6UDI3 P06489 Q88886 P05411 P03169 Q83950 Q6UDI1 P16799 P03233 P68272 P07299 Q6UDH0 P16735 Q6UDG8 P29832 P24436 Q9IG42 P89679 P03511 O10300 P28966 P25695 P28947 P24910 P0CA01 Q18LD4 A3DRP5 Q6UDJ3 P03091 P03222 P14984 Q77MS6 Q7TD09 P09294 P11265 Q9E6Q0 Q91EK1 O36381 Q6UDM2 Q9YNA4 P21061 P68578 Q5URA7 Q196W5 Q77MQ6 Q5UR16 Q5UQG0 P52585 A9Q1L1 Q5QGZ2 Q5UR81 P68677 Q82055 Q5UQ37 P34015 Q38653 A2T3Q0 P03154 P68570 Q5UR19 P08072

Dataset S2. The virus PEA entries for testing including 69 different proteins

Q6UDF5 Q65387 Q5MKM5 Q88940 Q88938 P09297 P22430 Q6S6Q7 Q6UDL7 Q9YMJ7 O39842 Q6UDI5 O39830 P11077 Q66677 P89462 P14971 Q6UDG5 Q77MS4 P04289 Q1HVH3 O36364 O92815 Q6WB95 P09290 P09613 P21049 Q83046 Q8JSZ3 Q89846 O55647 Q9IV52 O40976 P0C778 P03517 P04875 A5HBE1 P03589 Q5I146 P87607 Q76ZN5 Q00847 Q67684 Q65669 Q06A28 P08393 Q74125 Q91HK5 P07613 Q9IV54 P16761 Q9JFS4 P89460 O55265 Q89717 P04509 P26658 Q87039 P68559 P11317 P0C1K1 P03101 P27256 P04876 O55653 P03417 P16733 P52639 P01546

Dataset S3. The plant PNEA entries for training including 758 different proteins

Q3E936 Q9SH58 O64495 Q84JK8 Q9M2Z4 Q8VYE5 Q41350 Q8L7H3 A3CG83 Q4PR44 P32026 A3C5A7 P47925 P29062 Q9ZQI0 Q8RU27 P82900 P83880 P10496 Q9LD07 P43212 Q40375 P09789 P93218 P27483 Q01197 P10495 Q9MT28 Q8W4A5 Q9CA56

Q6Z2T6 B9TSP7 Q01402 Q94AR8 O23522 Q9LD57 P55826 Q7XBW5 B1VKF0 P38025
P0CE10 Q6AUR2 Q8H1T3 Q9LRN8 Q8L493 Q0V865 P32069 O22494 Q10P60 Q9FI53
Q9SCY3 Q66GP4 Q8RY73 Q5G1S8 O81908 Q7DM39 Q9M1X0 Q8GW64 O23278 Q9FMT1
O22795 Q9SA76 Q94KR8 Q9ZUU1 Q9LU68 Q9FJP3 A3BN26 Q8LDW8 O82768 O22506
Q9FGR7 O23365 Q9SQT8 Q9FLD8 Q93Z66 O80934 Q8VZF3 P93236 Q6AUK5 Q9LFG2
Q0WVV0 Q8W250 P42390 Q9FEA2 Q7XZF7 O80362 P42762 P83643 Q84LK7 Q93ZW0
Q8L633 A9SLE5 O22493 Q38929 Q43846 Q6K669 A2YXS5 Q05758 O65693 Q8LAP6
P42770 Q9STN5 Q0WTB4 Q6DBN2 Q8W593 O22553 O49434 Q940A6 Q0WNN7 Q9SZX3
Q9ZUC1 P56848 Q8W033 P0C7R2 Q9C550 Q9SZ52 Q7XPL2 O81304 Q9FJ82 Q02166
Q75LR2 Q9C5R8 Q9ZSS6 P55234 Q2RAR6 Q9SKP6 Q53NI2 Q84YK8 Q5NBJ3 Q6AV34
P46644 A8I2V9 Q69QJ7 Q9LMM6 Q93ZB6 O65567 O49204 Q9SIC9 Q9FKS0 Q9STK5
P93111 Q9C9C4 Q9LP37 Q6YZE2 P49572 Q9LFV0 Q109R6 Q944B6 Q5M755 Q9ASV6
Q6ZG77 P16180 Q84W65 B8B4H5 Q9SS80 P48417 Q6YYA3 Q9M4Y3 Q6Z398 Q5NAY4
A6H5M5 Q9SJ05 Q6ZIK0 P47924 Q9ZUU2 O23049 P25269 Q6NN02 Q8VXZ0 A2YS71
P0C565 Q9FV54 A9SCV9 Q7X9A0 Q42997 Q9FYC2 P69834 A2YM35 Q8LB01 Q43870
Q949X3 Q8GYL7 Q96266 Q8VY91 Q9LS25 A9S6X4 Q9FXG2 Q0IWL9 Q8LBZ4 Q5JMF2
Q9SJ20 Q9FKC6 Q8VZS8 Q56TU4 Q9SGE9 P93732 Q7XA48 P34066 P93733 Q9C5Z2
O03992 O22290 Q9FGZ9 Q93Y22 O22827 Q84TX2 Q8RY22 Q9SFC6 Q652Q8 Q6Z382
Q42563 A2ICC6 A2ZM73 Q9XHM1 P50651 Q6AUW3 Q9FJ70 O04379 Q5N749 Q9FJR0
Q9FMH4 Q9M9K1 Q9ZPI1 Q6Z844 A2YP56 O04350 Q1W375 Q9SV36 Q96321 Q8W4C3
P80969 O64765 A2ZIW7 Q9ZPV8 Q56ZI2 Q9FGY9 Q5PNU3 Q9ZTS1 A2WNB0 Q0WWQ1
Q9C827 A2X052 Q9ZPY7 Q8W1L6 Q9C7F5 Q53PC7 Q8L5U0 Q9LXC5 Q10LJ0 Q42510
A0MDQ1 Q9C744 Q96528 P56821 P43255 Q9SHJ0 P11573 Q93XK2 Q40271 Q7DLR9
Q9T034 O23717 Q9SS47 P46086 Q8H0T9 Q9T074 Q93WI9 Q8S926 Q7XJ96 Q9FJT8
Q8GZ45 Q9M9G7 Q9FIK7 O82200 Q8W207 O24210 B9HCR2 Q7XIZ1 O64527 Q9LD55
Q5JKN2 A2YR10 A2X635 P50217 Q9LMG8 Q8W1P0 Q9LNI2 Q9C566 A5BUU4 Q9AYP4
P52777 Q8VYU4 Q9SW95 Q2V0W1 P42158 P15252 Q9SLF3 Q6H6Q7 Q0E2Q3 O64564
Q84VA7 O65084 Q94HF1 O50044 Q9LST6 Q40392 D4AEP7 Q0J3D9 Q6R2V6 Q7G959
Q9LFA2 A2X0Q3 O80831 O82531 O04202 Q8L7G4 Q8L7S4 Q6NLV0 P80889 Q6L3Z4
Q6Z6G6 A2Q1V6 O04438 Q8S924 A2XY73 O50008 Q9FIZ7 A1L4Y2 Q9FF55 Q9STX5
P52885 Q5N8F2 Q9AS33 Q9STL5 Q6IWB2 Q6DUW8 Q6DUW6 Q6IWB1 Q3EDI6 Q0INM3
Q6IWA9 Q2V4E2 Q8LDN4 Q4ADV8 Q8S8N1 Q29PV4 Q9LV97 Q7XSN6 Q339K6 Q8L7W8
Q3E9I4 Q9LUA1 Q7SID5 Q3ECH9 Q7XUR3 Q9SJP6 Q9C9X5 Q6Z4G3 P94017 Q8H0W9
Q93YP4 P92513 A4RZ86 P38466 O49680 P38456 Q9M8M3 Q9FIT7 Q9SAH2 P93295
Q9LVD3 P08747 Q2QMG2 P92560 Q9S733 P93310 P92522 P10582 P38458 Q8L844
Q9LRP6 P93299 P10579 P92530 Q9SQT4 P92542 P92539 P92525 Q9SA60 Q9T0K7
P92540 P38464 Q9XGA0 P92543 P93317 P38455 Q0WM29 P92552 O81028 Q9LFM6
P38450 P92523 Q9M9X9 P92529 O22288 Q8LEZ4 Q9FGI6 Q9SDD6 O04904 Q04655
P38471 P92555 O24143 Q9SID3 P92537 P93293 P92515 P93294 P38463 P10581
Q94JX6 Q9SR86 Q9ZU27 P93308 P93282 Q04647 Q9FNE0 P93297 P93277 P93300
B8B7X6 P38469 Q0JM76 Q9FNG8 C5WU23 P92517 Q9SAK0 Q9C8J2 P92520 Q8L9A0
P38460 A2YQ56 P0C896 Q66GI4 P93309 Q9FH50 P92544 A2XNR6 P92566 P92534
Q9LUD6 P92561 Q5XET6 P93287 Q9SK66 P92554 P92516 P38474 Q9LR67 Q3ECH5
Q9SFV9 P92563 P38476 P10580 Q93ZY7 P38467 P92535 Q9ZU67 P38457 P38451

P92521 P92526 P92510 Q5JNC0 Q9SAA6 Q6Z4U2 P92548 Q9LER0 Q9LN22 P0C8Q6 Q94A16 Q9FX35 Q8LAH8 P93302 Q84J78 P92538 O22769 P38473 P92512 Q84VG6 Q6K9N6 P93281 Q9FME4 P93276 Q9FLJ6 P93292 Q9M316 P38468 Q3EAF8 O49354 Q9FS88 Q9CAA5 P93318 P38465 P93305 P92531 O80958 Q9SUC0 P38475 Q8W485 P92562 P92518 Q8LPF1 P38472 P92551 Q9SS83 Q9M0Y6 P42797 P38470 O49711 P92519 Q8VZC3 Q5XF75 P93291 Q9FZ24 P93315 Q940Q2 Q8L6Y7 P93301 P92524 Q9SZ67 Q38861 P13027 Q9SJF0 Q9FMT4 Q9SH88 Q9FG08 Q75IP6 Q93ZH2 Q8LI34 O80877 Q7X923 Q9FKH1 Q9C6V4 Q9ZQA8 Q6NPT8 O65418 Q8W4H1 O82422 O48653 Q8VZT0 A1YKY7 Q9FL62 A2WM14 Q6K5K2 Q9LU93 Q9LXE5 Q9C9F6 Q9C5V6 Q9SL02 Q9MA98 A9S0B8 Q9STF1 P58223 Q9C9S2 A2ZAI7 Q7XS74 Q9LYC1 Q39090 Q6L4L4 A9SVH7 Q9FLH0 Q6F6A2 A2YQU8 Q9FMU5 Q9ZRT1 Q9XEE6 Q9LUJ5 C0LU16 Q9ZRU9 Q9FL33 Q339W7 Q8L736 A8MQG7 Q6A332 Q8VYB1 P43333 P34881 Q9FDW0 Q9LME2 Q9FHB3 Q9C6I8 Q9LUG5 O23372 Q9M041 Q8L840 Q53HY2 Q7X7E9 P49030 Q8H1U4 Q84T61 Q682V0 Q9LUB6 Q27GK7 Q9LSN7 Q6ZIK7 Q2V3N5 Q84TI3 Q9SK39 O64687 A8JBB2 Q93WU8 Q0E3F8 Q00958 Q9M3G7 O65155 Q9C8Z4 Q9FE83 Q8LCQ3 Q9LIA4 O81037 O49279 Q9C9F4 Q9LKF9 Q9AWL7 Q8RWN7 Q9LXN4 Q9ZVH3 Q8L6Y4 Q7X9H9 Q8LQ36 Q8W234 Q9SVU7 Q9FGR0 Q94K01 B8B4D0 Q943I6 Q9ZPW2 Q8GY31 A2XW69 Q9M8K2 Q9SGE3 Q84K00 Q8W3M6 A2Y5G8 Q9C7A8 Q9LW85 Q9SVJ9 O04658 Q9SFD8 Q0WRC9 Q9SJ56 Q8LGU7 P38421 Q7X9B9 Q84JC0 A8ID74 Q93XX8 Q6PQQ4 A2XZI2 Q9FRH7 A2YX04 Q7XJS0 Q84W92 Q3ECP0 Q9AVK4 Q851R2 Q8W4M7 Q8H181 Q9STN3 Q8VY78 Q9FFF9 Q8L556 Q5NAN5 Q9FMV5 O65416 Q8RXF1 Q93Z00 Q5YGP8 A2YH41 Q39147 P0CAP5 Q9LZ03 Q0D9R7 B3DNN5 Q93ZL5 Q9LUQ9 Q9XGC9 Q9FJX0 Q9M2Q4 Q8RXK2 Q3EBZ2 Q8VY89 Q6E7D1 Q84MB2 Q9LEY9 O24160 Q9FWQ5 Q9T0C7 P24715 Q8LEF3 Q9FFX1 Q9SK02 Q8RWQ8 Q9LTJ8 Q9ATY5 Q0JKK6 Q94C11 Q6AWV1 Q9LS09 Q39290 Q42572 Q851V5 Q84L33 A2XFB7 Q9ZUB8 O48520 Q94AH9 O22165 Q5Z6B1 Q8VYP6 Q7X7L3 Q8LKW0 P35682 Q7F1M0 Q941D1 Q8VY05 Q84T68 Q9MBA3 Q9LHQ7 Q9ZP54 Q9LKI5 Q9ZNU6 Q38961 Q7XLX6 Q8S1Z1 Q9SLH0 Q0JP99 Q6EU10 Q9SHD0 O22899 A2YB34 Q9ZPV5 Q6ICX4 Q8GX29 Q9C578 Q682D3 Q570C8 O04420 O65201 B8AKX6 Q42807 P20347 Q02214 P59082 Q05047 P29059 P52407

Dataset S4. The plant PEA entries for testing including 261 different proteins

Q38967 Q9LYF6 O04714 Q8VZ80 Q8L796 Q9SZW4 Q9SSG3 Q9FY75 Q5PP12 Q9XIP7 Q9ZV07 Q9LUD7 O82811 Q93Z04 Q9FGS5 Q8LPK4 Q9LY23 A9SU70 Q84KJ7 Q3YL57 Q9LJD9 O48915 A1EC31 Q8S2W4 Q8VZQ5 P57752 Q9FMZ0 Q9LI83 Q42965 Q94F57 Q9LJN2 O24367 Q852F6 Q94EE4 Q9ZT17 Q94BZ1 Q5S1W2 Q8RX77 Q6NN00 A1XGB4 Q38998 Q6H3Z6 C5Y7C7 Q9SIA5 B8LQF9 P17816 O49432 Q8H112 Q08365 Q7Y618 P53535 P26259 Q9LJL3 Q9SL05 P11402 A0A332 Q9XH32 Q85X03 B2X1Z3 Q9FIG9 Q42536 Q9LQK7 Q94ID3 Q8LI30 B8APK3 O04603 Q9LV03 Q8GTZ9 P25864 Q84WN0 Q42805 P60315 Q41008 Q9SLD2 Q7PCJ6 Q8VYD4 B7FA90 Q2VEX9 Q32RT2 Q9M9Z2 P80680 P52768 Q9C5D0 Q8HUH2 Q40593 P31164 Q85BV1 Q94K73 P54773 Q8VY16 O23404 Q38932 P0CC48 P11891 Q9SN86 Q02028 B5BUZ8 Q9FNY0 A8CVF3 Q9SRQ3 O80448 Q08112 Q9CAJ0 Q8VZ59 Q43772 Q944C2 Q8S8I2 Q9SU30 Q0WVL7 O23702

P94077 Q940Z2 Q2V9B0 Q9M651 Q43125 P22200 Q9FNY2 P46259 Q9ZPQ3 P55925 Q8RXD9 B0EXJ8 P34788 Q8RWE8 Q9SBL1 Q9FFK1 Q7XA07 Q944A6 Q9ZUX7 Q9SR37 Q8VYP9 Q9FUY7 P48623 Q38798 Q8S8S1 Q8LFP1 Q93W54 Q0WPQ2 A2XWN6 Q0JL46 Q93Z29 Q9FK43 Q84WV9 Q56X72 Q9LZM1 Q94CG2 P33522 B9DHD7 Q68KI4 O04046 Q9ASU1 Q8L8W0 Q9XIE6 Q500W7 Q9FZE4 O49519 P85208 Q9S7T8 P82007 Q9M2T1 A7XDQ9 Q9SFE9 Q8VYU6 Q3EDG5 Q8L707 Q8S2T0 O80632 O48684 Q8L838 Q9FZD1 Q9SKC9 O81027 Q93YP7 Q9LVM1 Q9SIB9 Q9C641 P26870 Q9FJW4 Q1PE39 A8IW99 Q9M9M6 Q8LAD2 P42027 Q9FIJ2 Q9FMV1 Q07511 Q9LHI0 P29685 Q9ZPX5 Q8L7B5 P31839 Q9FNN5 P37900 Q8LDK3 Q7X9V3 Q9FNZ4 A9LNK9 Q6EVK6 Q9FKK2 Q9M658 Q9FKW0 Q0JL44 Q8H1U5 Q9LUA3 Q9C5Y4 Q9FJL0 D0EL35 Q9FQ19 Q9XI36 Q941Q3 Q9SU25 O04425 Q9FPW4 Q9SYK4 Q9M086 Q9FQ03 Q8GSA7 Q9LYD9 Q9SKZ1 Q9SY59 Q501D5 Q7XPY1 Q9LMA8 Q9SY69 Q8LPQ9 Q8LL04 Q9LRZ3 Q9FNZ5 Q9LG97 Q84VG7 Q5D869 Q6R0H1 Q9FLT2 Q708Y0 Q9LK40 Q2PS26 Q9SJQ6 Q6NLW5 Q9LME6 Q9FGW9 Q8VZD4 Q8LPS1 A1YGK9 O99010 Q6RH25 A7M935 O78678 A7M933 Q9SH59 Q9XGM1 P0CAN7 Q8H1G3 Q6MWE5 Q84ZM7 Q7XU31 Q9SYG9 Q6ICY4 P86795 O23654 Q8RWN2 Q9LS46

Dataset S5. The Gram-negative bacterium PNEA entries for training including 248 different proteins

P45763 B1VC86 Q6FYW0 Q46832 P15753 P65410 P45059 P36678 P24690 Q8ZBZ2 A5UAX6 Q46833 P59588 P45757 P25060 P34026 A9GI17 P55892 P34027 P31708 P44469 Q6FYW7 Q9K0U9 Q83C69 Q48253 P29041 Q7VSX5 Q60B79 Q8G0Y6 Q1RKL1 Q9JZN9 P34750 Q01244 P25733 B0UUL1 P45758 A5VW32 P13509 P45384 Q52657 Q9JYV5 Q1REC0 A1A7M1 Q9KKA3 P44600 P0A909 Q4QNS5 Q9HZY8 A1JQE6 Q9KRJ0 Q92IC3 O25021 P27474 P58598 A8W969 P0C6C5 Q8RSY9 P77790 A3UNN4 Q52481 O30916 A2S1Q1 A7FJ88 B4SX34 P35648 P17452 Q9HI36 Q56136 Q2SFU6 A2S1Q7 P0A1J5 Q8K9K0 Q9I596 A2PU44 Q8XZR1 Q2T711 Q03946 P55117 D2AJU3 P43948 P18773 Q9K323 P37050 Q52420 Q9FCZ8 P37928 P46007 P75715 P43319 P31058 Q60153 P53510 P43663 P77588 P45995 P42913 P37922 P25734 P25731 P26318 A5UEK8 B1K4Q5 Q1I490 Q65R92 A5F7I0 A5UDB3 B0UU63 Q7VU58 P55601 Q665F0 B9L8L4 Q2YPY5 P55691 P33590 Q8ZE65 P36267 Q56797 Q9KU25 P23716 Q2W0J7 Q59094 A8AFT6 P0AF86 P21175 Q01578 Q8ZD92 Q0WFT9 A5VU57 P33362 P0A3Z5 Q4UNH3 Q51371 P59788 P76108 P44526 P44542 P55669 Q8XUU7 Q7VU12 P54083 P21258 Q46863 P16028 Q48KB0 P39906 P76128 P46448 Q083B9 O52376 P28722 B2U9Y9 Q66D46 P12634 P21408 Q87UY0 Q7VR84 A5VU91 Q47537 Q1CHD5 Q7VR38 A4W8D7 P39187 P24735 P25549 P55401 O08430 P0AFX9 P45016 A1A9U1 B6JM17 Q9I4P4 P28607 Q21K25 Q4K4H3 Q52947 P55568 A4TPX1 P45017 Q8X6H3 Q8EEQ7 Q9Z3R5 P55659 Q9Z3Z9 P50500 Q9KPW1 P16682 P14774 P76042 Q2YLW8 Q46636 P96156 P28249 P46458 P45766 P0AF82 C7C422 P55454 P37648 Q59635 P0ADU5 Q9I4U2 P26918 P57418 P44764 Q52825 P71391 P55417 Q50926 P45028 A1JMV0 Q6FYW2 Q887Q8 P04129 Q887Q0 P45168 Q7MA16 P45161 Q87C91 P52700 P26365 P06610 P76193 Q02728 Q6CZ31 P70791 P39370 P75853 Q50940 Q0T6D2 P35120

P0AG79 P44566 P75687 Q1LSS0 P44654 P75694 Q31YK5 P58358 Q9KGS7 Q46444 Q52309 P78067 Q44009 Q2G9L1 O06875 Q7MD15 P45285 P69965

Dataset S6. The Gram-negative bacterium PEA entries for testing including 207 different proteins

Q01248 A5VVQ3 O07899 P0A8Z3 Q16DZ2 Q163Y8 A9KFZ1 Q1R003 P57037 P31601 A9IZD8 P31896 A5WD44 Q0A8Z4 B2I8J9 P64604 Q2NQH3 Q00184 P43799 Q4QMJ5 Q03583 Q59661 A5WG48 A9IS13 P52106 P32696 Q9KU26 P45408 A1URA3 P0AFN2 Q9PNJ9 P0ADA3 Q8XU11 Q87KW0 B1JS04 Q2LXE8 Q03476 Q9I424 Q6D8S7 D5ARG8 Q8XZI4 P58380 Q87EJ9 Q1QD88 Q9WX70 Q7VNT5 Q4K884 A1VZK8 Q1DB00 P57038 Q0BHC9 P44332 P44704 D5AQY8 Q7VRA5 O54245 A1B0E4 P11460 Q4UND5 Q9KPS2 Q12FJ6 B0T335 Q9X772 A1SS94 B0T0X6 A7H688 Q3K4T4 Q2IL15 A3M7G4 B7V5T6 C9Y1J4 Q06277 B5FGS5 A1IPF8 P46130 P06970 P75906 P52143 P09181 A8FM78 P0A0V0 A1JQ26 P58042 O82861 P0A1K5 P77196 Q08868 P75820 P05351 P15484 C4K7Y9 P0AC02 Q7VKB7 Q01567 A1VXG4 Q46668 P55969 Q05098 Q45977 Q9K0V0 P69434 A7MSG2 P33410 P45420 Q02104 Q6FYW8 A4WEW2 A5F383 P64467 P0A0Z7 P23537 Q8Y199 Q89AM4 P0A7X6 P44443 Q9JW39 P18319 P0A9V1 A0Q5J9 P0AF26 P0ADS2 P27236 Q2K859 Q57BR6 P76344 Q7M8Y8 Q87D45 Q84H44 P23871 Q05770 P44818 P0AG86 Q8UGK1 P40290 P44539 Q9HTK8 P0AAT6 Q52964 Q9HUF7 P0A1N2 P31572 Q5NR44 P27247 P0AGK4 P0A898 Q72CB8 P07363 P00956 Q6LVI6 A5WC58 P0A8X0 Q04640 Q05205 P04981 P24419 Q87P32 Q7BVH7 Q5ZRQ0 P24153 P0C8Z8 Q8ZQ59 P07268 D0ZI38 O84947 D0ZXR5 P08538 P0A3W8 Q8Z6A7 Q8RSY1 Q7PC62 P24791 Q9R803 P45355 P38441 P0A3R5 P24216 P01556 P26877 P04979 P18914 Q8ZQC8 Q8KQN8 Q47278 D0ZPH9 P14608 D0ZIB5 Q07259 P42188 O68609 A1YYW7 Q63K42 Q05JY7 P0C1A5 P45996 Q59589 P42186 P42187 P23857 P76341 P19573 P14775 Q58AD3 P37690 P69741 Q72EC8 P24702 P00805
